# Establishment and genetic characterization of cell lines derived from proliferating nasal polyps and sinonasal inverted papillomas

**DOI:** 10.1038/s41598-021-96444-y

**Published:** 2021-08-24

**Authors:** Thawaree Nukpook, Tipaya Ekalaksananan, Tohru Kiyono, Pornthep Kasemsiri, Watchareporn Teeramatwanich, Patravoot Vatanasapt, Surachat Chaiwiriyakul, Piti Ungarreevittaya, Jureeporn Kampan, Kanha Muisuk, Chamsai Pientong

**Affiliations:** 1 Department of Microbiology, Faculty of Medicine, Khon Kaen University, Khon Kaen, Thailand; 2 HPV & EBV and Carcinogenesis Research Group, Khon Kaen University, Khon Kaen, Thailand; 3 Project for Prevention of HPV-Related Cancer, Exploratory Oncology Research and Clinical Trial Center, National Cancer Center, 6-5-1 Kashiwanoha, Kashiwa, Chiba 277-8577 Japan; 4 Department of Otorhinolaryngology, Faculty of Medicine, Khon Kaen University, Khon Kaen, Thailand; 5 Department of Pathology, Faculty of Medicine, Khon Kaen University, Khon Kaen, Thailand; 6 Department of Forensic Medicine, Faculty of Medicine, Khon Kaen University, Khon Kaen, Thailand

**Keywords:** Cancer models, Head and neck cancer

## Abstract

To better understand the pathogenesis of nasal polyps (NPs) and sinonasal inverted papillomas (SIPs), we aimed to establish cell lines from fresh tissues of NPs and SIPs and characterize them. Primary cell cultures were obtained from two NP tissues (NP2 and NP3) and one SIP tissue (IP4). All the cells were polygonal in shape, expressed cytokeratin 14, and had normal diploid chromosome status. HPV58 DNA was detected in NP3. To obtain immortal primary cells, NP2 and IP4 cells were transduced with a combination of mutant CDK4, cyclinD1 and TERT. These cells were thereafter named NP2/K4DT and IP4/K4DT, respectively. HPV58-positive NP3 cells were transduced with TERT alone, the resulting cells named NP3/T. Phenotypic and genotypic identity of original tissues and derived cells was investigated. All the cell cultures with transgenes were confirmed to be derived from their parental cells and primary tumor tissues by analysis of short tandem repeats (STR) and maintained in vitro growth, genetic profiles and gene expression characteristics of the primary cells. These virtually immortalized cells, as well as the primary cells, have potential as in vitro models for studying the pathogenesis of NPs and SIPs and for preclinical study to develop new therapeutic agents.

## Introduction

Nasal polyps (NPs) are common, but benign, hyperplastic growths of the nasal mucosa^1^. Patients with NPs might be asymptomatic or might have various nasal problems such as nasal obstruction, rhinorrhea, and nasal congestion^2^. NPs frequently recur and are mostly associated with chronic rhinosinusitis^1^. Even though they are common, their etiology and pathogenesis are poorly understood^3^. Several causes of NPs have been proposed such as inflammation, neoplastic origin, bacterial infection, and fungal infection, but the precise mechanisms are still under investigation^4–8^. There is no effective treatment for NPs: treatment strategies only focus on the reduction of NP symptoms by using medical or surgical approaches according to the inflammatory or neoplastic origin of the NPs^4^. Despite attempts to define a core set of biomarkers that are related to NP pathogenesis to improve treatment outcomes, only limited progress has been made^9–12^. More research is needed to elucidate underlying molecular mechanisms of NP pathogenesis and to develop new therapeutics.

Sinonasal inverted papillomas (SIPs) are benign tumors of the nasal cavity and paranasal sinuses^13,14^. SIPs are characterized by the inward growth of epithelial cells into the underlying supportive tissue^15^. It is the second-most common benign tumor of the sinonasal tract, representing approximately 0.5–4% of all primary nasal tumors, and is more frequently found in males than in females in the fifth and sixth decades of life^16^. The etiology and pathogenesis of SIPs remain unclear^17^. Moreover, SIPs frequently recur following treatment and can cause local destruction^18^. A relationship between SIPs and sinonasal squamous-cell carcinoma (SNSCC) has been reported^19,20^. The SIP treatment of choice is surgical removal of the tumor mass: its success depends on complete resection of the entire tumor. Preoperative medical treatment is used to reduce existing inflammation and intraoperative bleeding to improve conditions for surgery. In some cases, such as SIP-associated carcinoma if surgery is impossible, radiation therapy might be considered^21^. A review of surgical outcomes of 36 SIP cases spanning 17 years reported a recurrence rate of 13.3% and 16.6% of cases who underwent endoscopic and combined/open approaches, respectively^22^. Despite improved treatment strategies, the recurrence rate of SIP is still high, and new, alternative, treatment strategies are required. Due to the rarity of this disease, there is limited information on its characteristics and treatment outcomes. An in vitro model might be a good system in which to study SIP etiology, biology and pathogenesis.

More studies on NPs and SIPs are required to understand their pathogenesis. Cell lines have been widely used as in vitro models for research into tumor biology^23^. They are useful for the study of the genetic and epigenetic basis of many diseases and excellent models for understanding a range of biological mechanisms including tumorigenesis. Examples include the investigation of deregulated genes, proteins and signaling pathways during disease initiation and progression. Moreover, cell lines can be used for the development of new therapeutic strategies and preclinical testing. In this study, we aimed to establish new sinonasal cell lines from NP and SIP tissues to characterize their chromosomes and genetic alterations. Three new primary sinonasal cells and their derivatives were established from two NPs and one SIP tissue. They should be useful in vitro tools for studying the pathogenesis of NPs and SIPs and for preclinical testing of new therapeutic agents.

## Results

### Characteristics of primary cells

**Figure 1:**
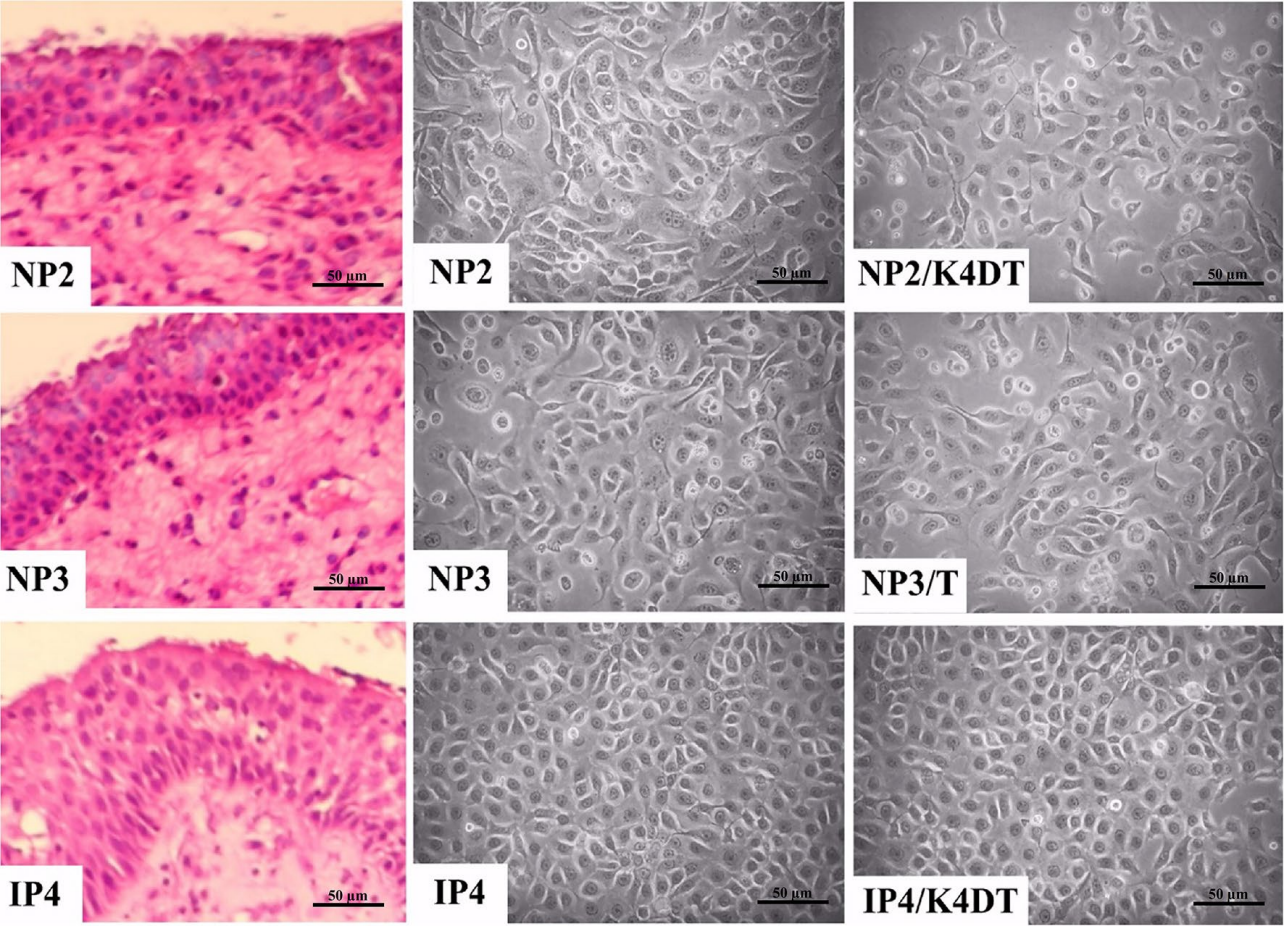
Photomicrograph of a representative H&E stained paraffin section of the original primary tumor (left), and the polygonal morphology of established primary cells (middle) and primary immortal cells (right). The primary cells, NP2, IP4, and NP3 were transduced with a combination of CDK4R24C, cyclin D1, and TERT or TERT alone, respectively, to obtain primary immortal cells. All established cells were cultivated in FYAD-medium in cell-culture flasks and incubated in 5% CO_2_ at 37 °C. At the 70–90% confluence, all established cells showed a similar morphological character to their primary tumor cells. Images were taken under the microscope with 200 × magnification (scale bars: 50 µm).

**Figure 2:**
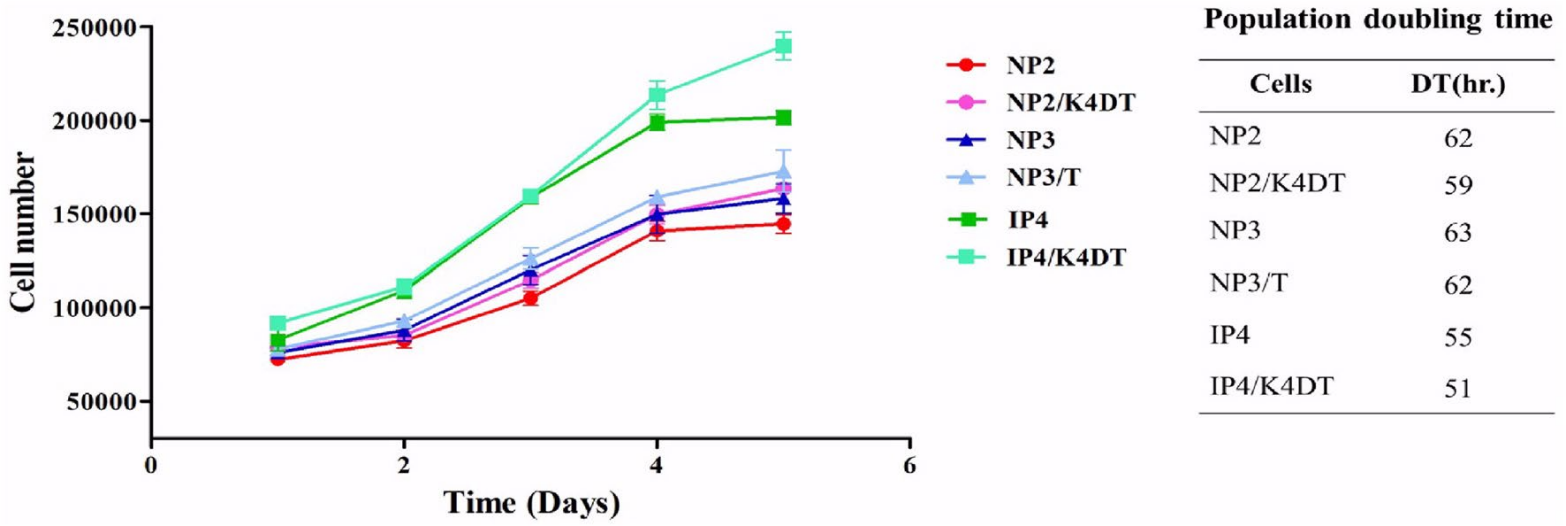
Growth curves and population doubling time (DT) of NP2, NP3, and IP4 cells and their derivatives. Viable cells of the triplicate wells were counted every 24 h for 5 days as described in the Methods, and the mean and the SD were plotted (left panel). Population doubling time was calculated during the exponential growth phase as described in Methods (right). Note that IP4 and IP4/K4DT cells derived from an inverted papilloma showed a faster proliferation rate than NP2, NP2K4DT, NP3, and NP3/T cells derived from nasal polyp cells (*p* = 0.0227). DT; population doubling time.

Primary sinonasal cells were obtained from fresh tissues of two NP patients named NP2 and NP3, and from one SIP patient named IP4. All the cells grew as monolayers in cell-culture flasks and were polygonal in shape, as shown in Fig. 1. The population-doubling time ranged between 55 and 63 h: IP cells grew faster than NP cells (Fig. 2). NP2, NP3, and IP4 cells could be subcultured until passages 13, 12, and 13, respectively.

HPV DNA was detected in NP3 cells, and reverse line-blot hybridization (RLBH) revealed the presence of HPV58 DNA in this case. NP2 and IP4 showed no evidence of HPV infection.

### Immortalization and primary immortal cell characteristics

**Figure 3:**
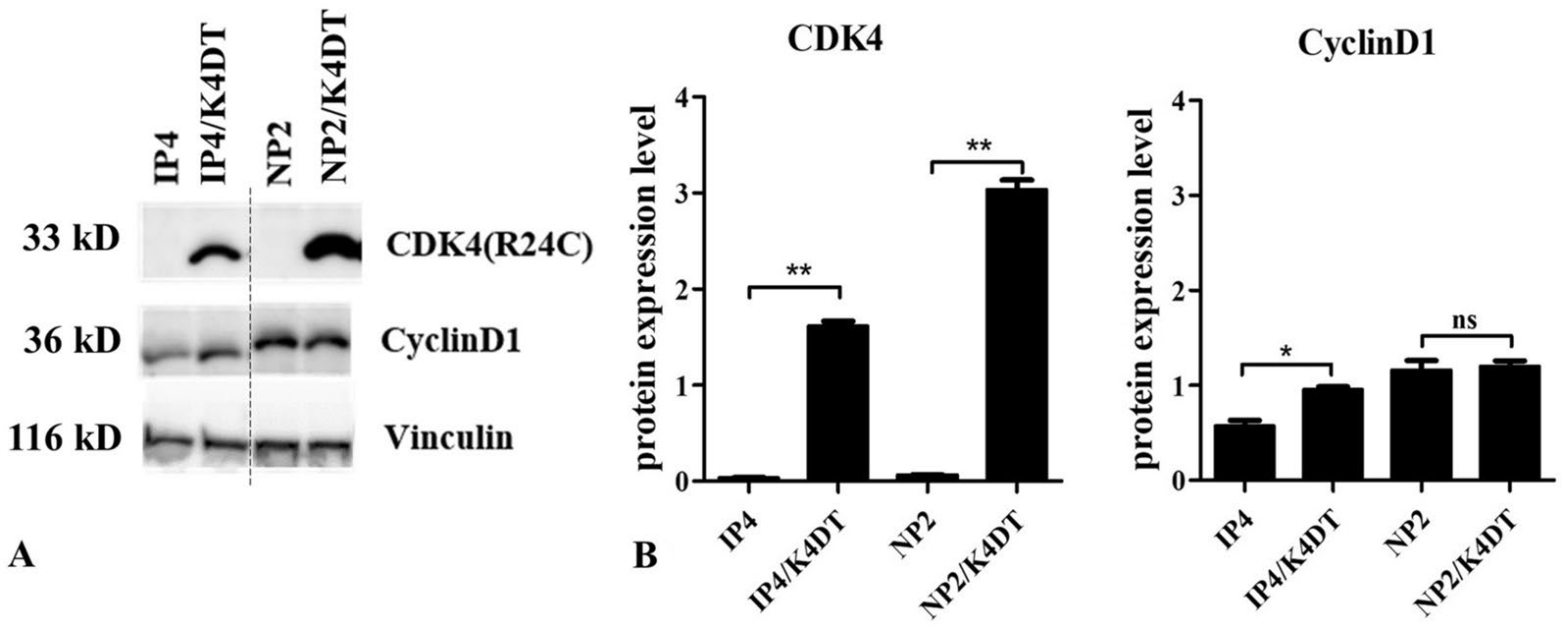
Transgene expression in IP4/K4DT and NP2/K4DT cells. (**A**) Expression levels of CDK4 and cyclin D1, as well as vinculin as a loading control in IP4 and NP2 cells and those transduced with CDK4R24C, cyclin D1, and TERT were examined in triplicate by western blotting. (**B**) Band intensities of CDK4 and cyclin D1 (normalized against those of vinculin) were quantified and indicated as bar graphs. Asterisks indicate statistically significant differences (*p < 0.05; **p < 0.01), and “ns” indicates not significant.

For the immortalization of normal human epithelial cells, both inactivation of the p16INK4A/pRB pathway and telomerase activation are required^24^. Therefore, to obtain primary immortal cells, NP2 and IP4 cells were transduced with cyclin D1 and mutant CDK4 (CDK4R24C: a p16INK4A-resistant form of CDK4), and human telomerase reverse transcriptase (TERT). In HPV58-positive nasal cells (NP3), pRb is considered to be inactivated by HPV58 E7 proteins, therefore, NP3 cells were transduced with TERT alone. As expected, TERT alone efficiently extended the lifespan of NP3^25^, and the cells were renamed as NP3/T. The combination of cyclin D1, CDK4R24C, and TERT also efficiently extended the lifespan of the HPV-negative nasal cells, NP2 and IP4, and these lines were named NP2/K4DT and IP4/K4DT, respectively. Vector-transduced control and uninfected control cultures stopped growing around passages number 10 to 11. The expression of transgenes, cyclin D1 and CDK4R24C were confirmed by western blotting (Fig. 3, supplementary). Cell morphology of NP2/K4DT, IP4/K4DT, and NP3/T was similar to that of their parental cells. The doubling times of all transduced cells were similar, but a little bit shortened compared to their parental cells (Fig. 2). At the time of writing, the current passage numbers for NP2/K4DT, NP3/T, and IP4/K4DT were 24, 23, and 30, respectively. We consider it likely that they were virtually immortalized though they have not reached 100 population doublings.

### Short tandem repeat (STR) analysis

The STR analysis of the established primary and primary immortal cells was analyzed and compared to STR profiles of primary tumor tissues. According to the personal information protection law guidelines, not more than nine profiles of STR loci from each sample can be shown (Table 1): data from the remaining 15 loci are not shown. The established primary and primary immortal cells share similar markers and match with the STR profiles of the primary tissues obtained from the patients. These results supported their identity and confirmed the same origin of the cells.

**Table 1:** STR profiles of primary and primary immortal cells compared to primary tissues.

	NP2			NP3			IP4		
Loci	Tissue	Primary cells	Immortal cells	Tissue	Primary cells	Immortal cells	Tissue	Primary cells	Immortal cells
D3S1358	16, 16	16, 16	16, 16	16, 16	16, 16	16, 16	16, 16	16, 16	16, 16
vWA	14, 17	14, 17	14, 17	14, 17	14, 17	14, 17	14, 17	14, 17	14, 17
D16S539	10, 11	10, 11	10, 11	11, 12	11, 12	11, 12	12, 14	12, 14	12, 14
CSF1PO	10, 10	10, 10	10, 10	11, 11	11, 11	11, 11	11, 12	11, 12	11, 12
D6S1043	12, 17	12, 17	12, 17	12, 13	12, 13	12, 13	13, 13	13, 13	13, 13
D8S1179	10, 13	10, 13	10, 13	14, 15	14, 15	14, 15	10, 15	10, 15	10, 15
D21S11	30, 31.2	30, 31.2	30, 31.2	32.2, 32.2	32.2, 32.2	32.2, 32.2	30, 31	30, 31	30, 31
D18S51	15, 15	15, 15	15, 15	12, 15	12, 15	12, 15	19, 21	19, 21	19, 21
AMEL	XY	XY	XY	XY	XY	XY	XY	XY	XY

### Chromosome analysis

**Figure 4:**
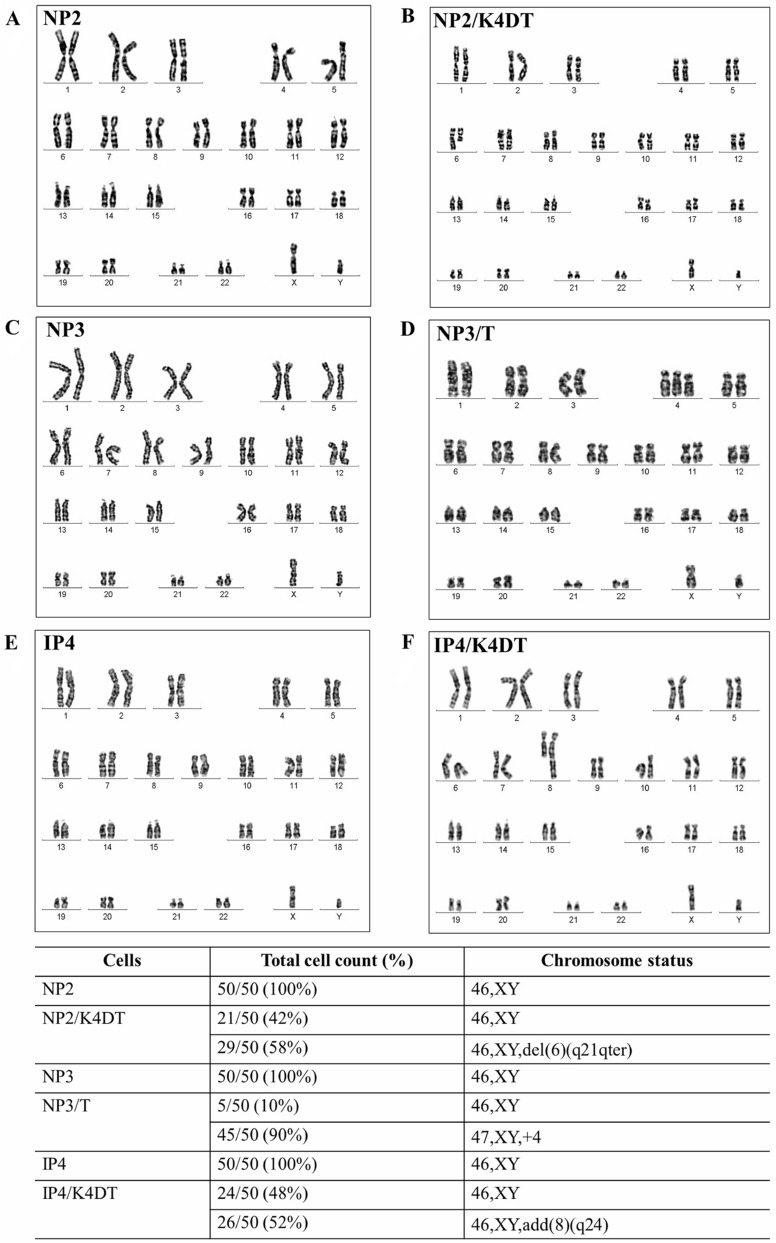
Karyotype variation of primary immortal cells compared to their primary cells. Karyotypes of NP2, NP2/K4DT, NP3, NP3/T, IP4, and IP4/K4DT were analyzed as described in Methods. The normal diploid chromosomes of primary cells (**A**,**C**,**E**) and representative images of abnormal karyotypes for NP2/K4DT (**B**), NP3/T (**D**), and IP4/K4DT (**F**) were presented. The results are summarized in the bottom table.

The chromosomal karyotypes of all established cells were analyzed. Results demonstrated that the NP2, NP2/K4DT, NP3, NP3/T, IP4, and IP4/K4DT cells had human male karyotypes. All primary cell cultures (NP2, NP3, and IP4) had normal diploid chromosomes and did not show gains or losses of regions in chromosomes. Some chromosomal abnormalities were observed in some populations of primary immortal cells. In NP2/K4DT cells, there was a partial deletion of chromosome number 6 in 58% (29/50) of the cell populations. Chromosomal addition was found in some populations of NP3/T and IP4/K4DT. In NP3/T, 90% (45/50) of the cell populations exhibited trisomy chromosome number 4, whereas a portion was added to chromosome number 8 in 52% (26/50) of IP4/K4DT cell populations as shown in Fig. 4.

### Genetic mutations analysis

**Figure 5:**
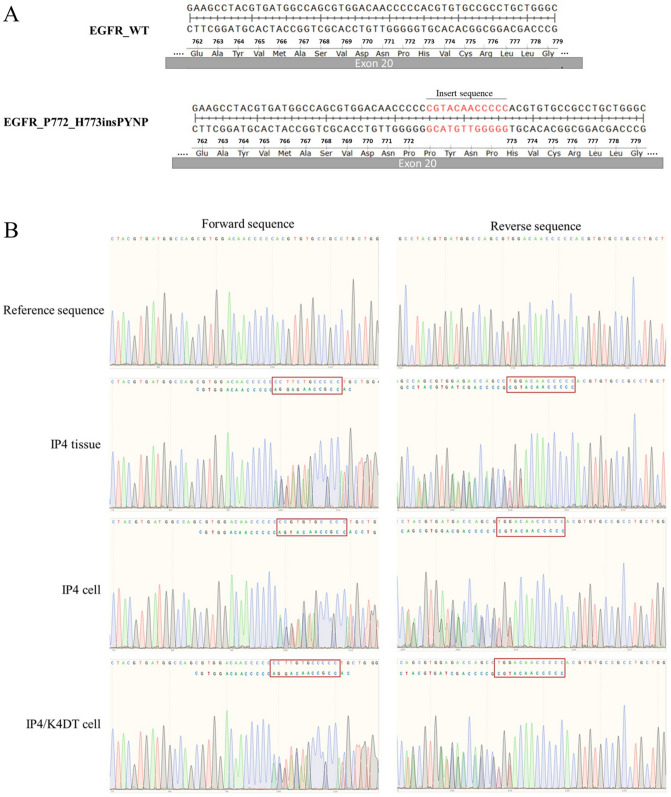
EGFR exon 20 insertion mutation in IP4 tissue and its derivatives. Mutation of EGFR exon 20 was analyzed by direct sequencing as described in Methods. (**A**) Schematic of wildtype- (Top) and IP4-derived EGFR exon 20 nucleotide and the corresponding amino acid sequences (Bottom). (**B**) EGFR exon 20 sequence electropherograms of IP4 tissue and its derivatives. The electropherograms were analyzed by CRISP-ID and the Mixed Sequence Reader. Both analyses identified the same heterozygous insertion mutation of EGFR exon 20 as indicated in (**A**) in both cell lines as in the primary tumor.

**Figure 6:**
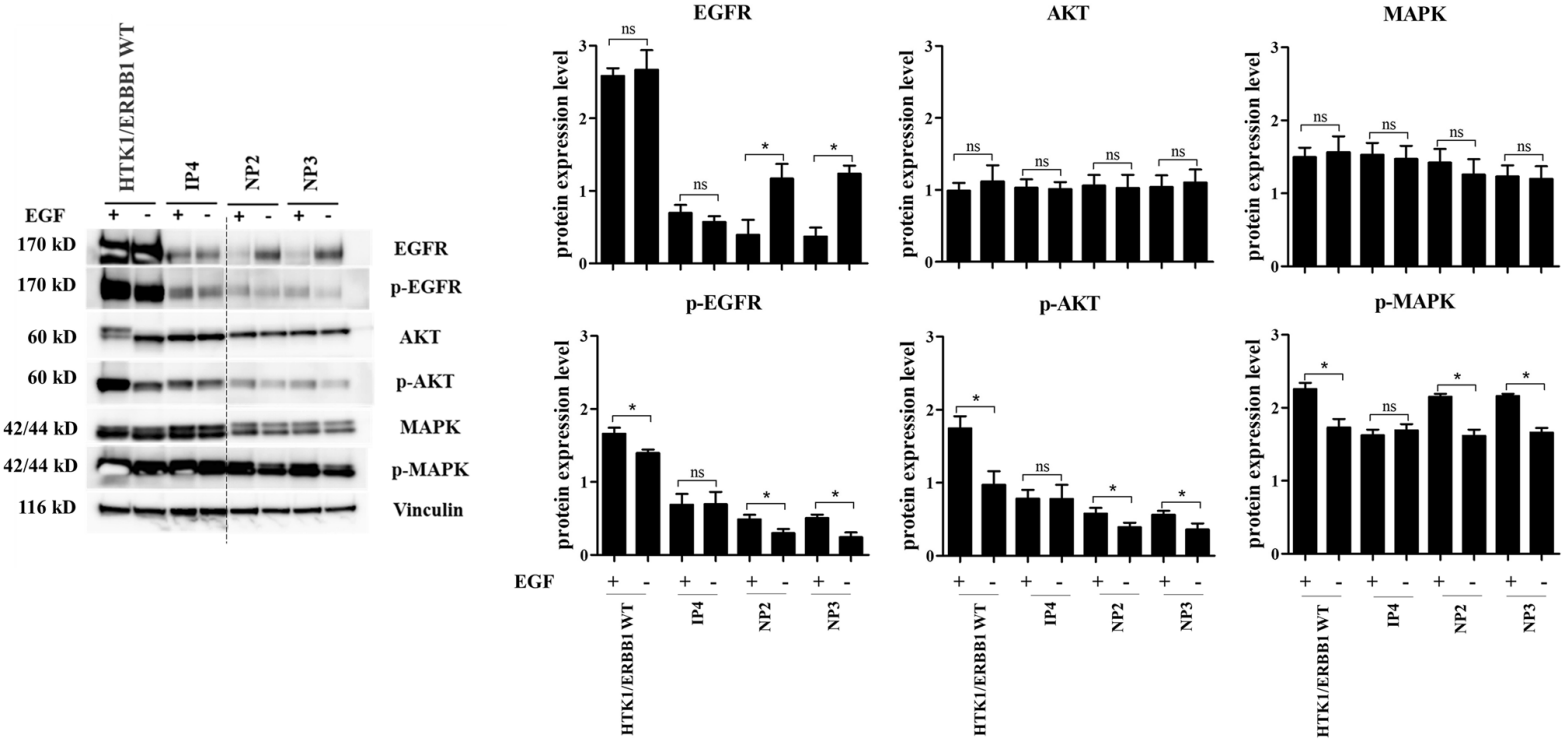
Constitutive activation of the EGFR signaling pathway in the IP4 cells harboring the P772_H773insPYNP mutation in EGFR exon 20. The cells were cultured in triplicate 6-well plates, starved overnight in serum- and EGF-free FYAD medium, then incubated in the presence or absence of 50 ng/ml of EGF for 30 min prior to harvesting cell lysates and analyzed by western blotting. The total and the phosphorylated levels of EGFR, AKT, and MAPK were detected by the antibodies described in Methods (left panel), and the intensities of individual bands were indicated by bar graphs (right panels). Note that the phosphorylated levels of EGFR, AKT, and MAPK were increased in both presence and absence of EGF in culture medium in IP4 cells. Asterisks indicate a statistically significant difference (*p* < 0.05), and “ns” indicates not significant.

Various genetic alterations have been found and associated with tumorigenesis. Mutation of TP53, EGFR (ERBB1), KRAS, and PIK3CA are frequently observed in NP and SIP. Therefore, mutations of these genes at known mutation hotspot sites were investigated by sequencing. No mutations were found in TP53 (exons 5–9), KRAS (exon 2), and PIK3CA (exons 9 and 20) in any cells. In EGFR (exons 19 and 20), only IP4 and IP4/K4DT had an insertion mutation of 12 nucleotides (CGTACAACCCCC) in exon 20 leading to an insertion of four amino acids between amino acid residues N772 and H773 (Fig. 5). This mutation in IP4 was novel and named P772_H773insPYNP (Table 2). This mutation results in ligand-independent activation of EGFR as shown in Fig. 6, supplementary.

**Table 2:** Characteristics of the established sinonasal cells.

	NP2	NP2/K4DT	NP3	NP3/T	IP4	IP4/K4DT
Cell morphology	Polygonal	Polygonal	Polygonal	Polygonal	Polygonal	Polygonal
Population doubling time (hr.)	62	59	63	62	55	51
HPV infection	No	No	Yes (HPV58)	Yes (HPV58)	No	No
**Genetic analysis**						
TP53: exon 5–6	No	No	No	No	No	No
exon 7–9	No	No	No	No	No	No
KRAS: exon 2	No	No	No	No	No	No
PIK3CA: exon 9	No	No	No	No	No	No
exon 20	No	No	No	No	No	No
EGFR: exon19	No	No	No	No	No	No
exon20	No	No	No	No	Yes (P772_H773insPYNP)	Yes (P772_H773insPYNP)

### Expression of EGFR, p53, KRAS, PIK3CA and cytokeratin 14

**Figure 7:**
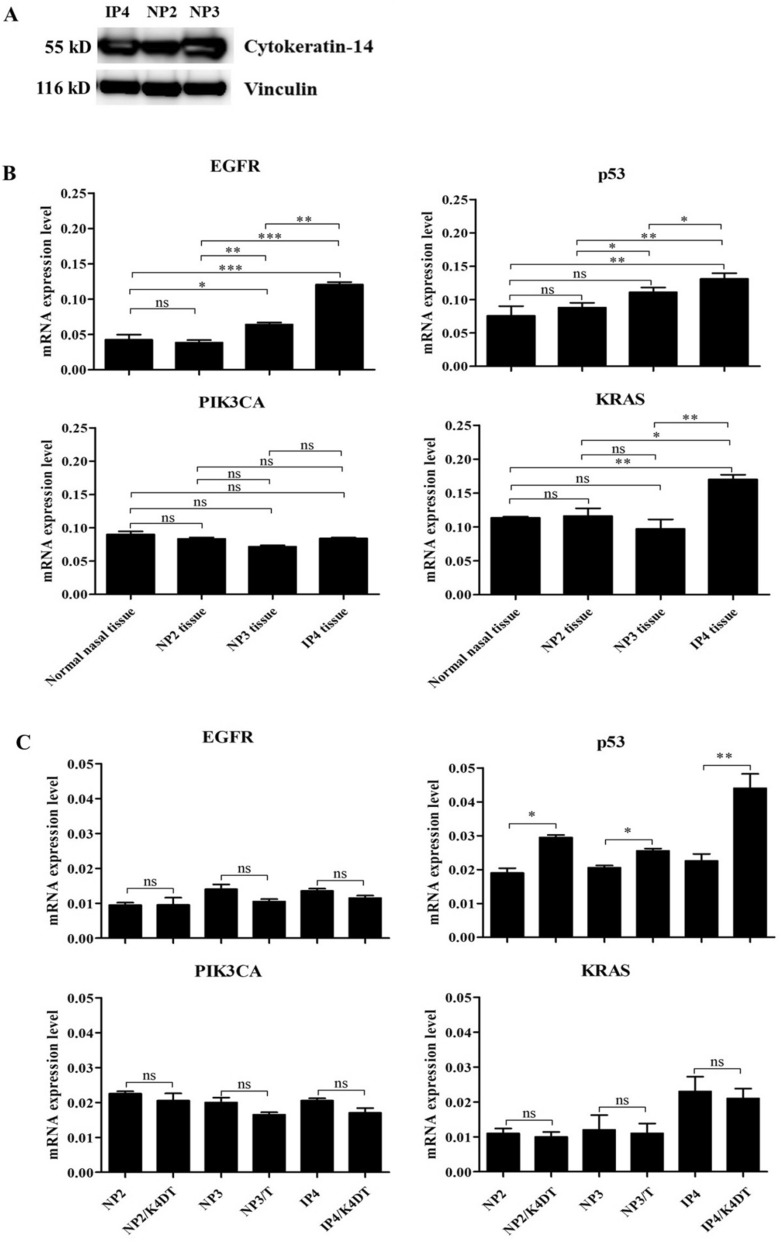
Epithelial origin of established primary cells and the mRNA expression level of EGFR, p53, KRAS, and PIK3CA in primary tumor tissues and established cells. (**A**) Total cell proteins prepared from NP2, NP3, and IP4 cells cultivated in FYAD medium were analyzed for cytokeratin 14 as well as vinculin as a loading control by western blotting. (**B**,**C**) Total RNA was extracted from primary tumor tissues (**B**) and established cells (**C**) and analyzed for expression of several genes by RT-qPCR as described in Methods. Note that established cells showed a similar expression pattern of target genes to their primary tumors. Asterisks indicate statistically significant difference (*p < 0.05; **p < 0.01; ***p < 0.001), and “ns” indicates not significant.

To confirm the origin of the established primary cells, the expression of a squamous epithelium marker, cytokeratin 14 was investigated by western blot analysis. All established primary cells expressed cytokeratin 14, confirming their origin as keratinocytes as shown in Fig. 7A, supplementary. To investigate the gene-expression levels of EGFR, p53, KRAS, and PIK3CA in established cells and primary tissues, RT-qPCR was performed using specific primers. In tissue samples, the expression of PIK3CA was similar in all primary tissues. Higher levels of p53 were observed in IP tissue than NP tissues. Increased expression of KRAS was observed in IP4 tissue compared to others. Higher levels of EGFR were detected in HPV58-positive NP3 tissue and IP4 tissue compared to NP2 and normal nasal tissue as shown in Fig. 7B. In established cell cultures, a similar trend of gene expression level was observed as shown in Fig. 7C. For EGFR, PIK3CA and KRAS, gene expression levels were similar between primary and primary immortal cells in each case. There was increased expression of p53 in all primary immortal cells compared to their parental primary cells (Fig. 7C).

## Discussion

In the present study, we describe the establishment of cell cultures from two NP and one SIP derived from samples with different clinical characteristics (Table 3). All cells were grown in monolayers. IP cells grew faster than NP cells (Fig. 2). Our result corresponded with studies of Mumbuc S et al. and Guichard C et al. who demonstrated that the epithelial cell proliferation rate of SIP was greater than that of NP^26^ and that expression levels of cell cycle-related proteins, PCAN, Ki-67, and p53 were increased in SIP tissues to a greater degree than in NP tissues^27^. Upregulation of the proliferation marker, PCNA, is associated with an increase of p53 in NP relative to normal mucosa^28^. In agreement with a previous report^29^, our gene expression results showed higher p53 expression levels in SIP cells than NP cells (Fig. 7), though NP3 was HPV58-positive, implying expression levels of HPV58 E6 were not high enough to target p53 for degradation and to activate telomerase. The epithelial origin of all primary cell cultures was demonstrated by investigation of cytokeratin 14 expression (Fig. 7). HPV infection has been linked to some head and neck cancers and is frequently detected in tumors with intact TP53^30^. HPV infection has been observed in up to 40% of NP^31^ and about 37.8% of SIP cases^32^. HPV, therefore, seems to be associated with the development of NP and SIP, but its role in this development remains undetermined. Two established cells, NP3 and NP3/T, were positive for HPV58, whereas no HPV-DNA was detected in the other NP and IP cells (Table 2).

**Table 3:** Patients and tumor characteristics.

	NP2	NP3	IP4
Age (year)	19	11	68
Gender	Male	Male	Male
Diagnosis	NP	NP	SIP
Tumor origin	Maxillary	Maxillary	Nasal cavity (middle turbinate)
Staging	Grade III	Grade III	Krouse staging T4
Eosinophil count	Low (9/HPF)	High (50/HPF)	–
Dysplastic change	–	–	Mild
Recurrence	Yes	No	Yes
HPV	No	Yes (HPV 58)	No
EGFR mutation	No	No	Yes (P772_H773insPYNP)
Pre-treatment	Intranasal corticosteroid and antihistamine	Intranasal corticosteroid and antihistamine	None
Post-treatment	Intranasal corticosteroid and antihistamine	Intranasal corticosteroid and antihistamine	None

In an ordinary culture system, the lifespan of primary cells is finite. After a certain number of passages, the primary cells undergo irreversible cell-cycle arrest called senescence^33^. Previous study has shown that HPV16 E6, which activates TERT, and E7 which inhibits pRB, is sufficient to extend the lifespan of cells^34^. However, the ability of E6 to activate TERT varies among high-risk HPVs, and in some cases, E6 and E7 might not be sufficient to immortalize primary epithelial cells. Therefore, the addition of TERT into the combination of E6 and E7 could be sufficient to immortalize the cells^24^. In the current study, the primary HPV58-positive cells, NP3 became senescent at about passage 12. We therefore tried to immortalize them by transduction of TERT into the cells, which increased lifespan of the cells, leading to generation of NP3/T. Transduction of TERT in combination with mutant CDK4 and cyclin D1 is another technique to induce primary cells to become primary immortal cells. The transduction of TERT into primary cells results in restoration of telomeres, while transduction of mutant CDK4 and cyclin D1 inactivates pRB by phosphorylation leading to enhance cell proliferation and cell-cycle progression without compromising differentiation potential^35^. In human-derived corneal epithelial cells, forced expression of mutant CDK4 and cyclin D1 together with TERT could induce immortalization while retaining the characteristics of the primary cells^36^. These results indicate that the cell-cycle regulator networks are associated with cell-cycle arrest, especially p16/Rb signaling pathways^37^. In the current study, the primary HPV-negative nasal cells, NP2 and IP4, become senescent at about passage 13. To immortalize them, TERT in combination with mutant CDK4 and cyclin D1 were transduced into the cells, leading to generation of NP2/K4DT and IP4/K4DT.

STR profiles of primary and primary immortal cells were compared to STR profiles of the primary tumor tissues. Results confirmed their individual origin (Table 1). Previously, cellular immortalization using mutant CDK4 and cyclin D1 in combination with TERT (K4DT) could generate immortalized cells with an intact chromosomal condition such as in human dental pulp stem cells, PT-5025^38^. However, this system sometimes generated immortalized cells with chromosomal abnormality. In immortalized cells (using the K4DT system) from the Bonin flying fox (*Pteropus pselaphon*), monosomy of chromosome 14 was observed in 90% (45/50) of the cell population^39^. In human ovarian surface epithelial cells, chromosomal instability was found in 8% of immortalized cells, HOSE1C^40^. Therefore, the chromosomal status of each established cell cultures was investigated to explore the effect on the chromosomal stability of transgenes used for generating primary immortal cells. Karyotypic and G-banding results revealed that all the primary cells examined maintained normal diploid appearance. However, abnormal karyotypes were found in some populations of the primary immortal cells. A part of chromosome number 6 was deleted (46,XY,del(6)(q21qter)) and chromosome number 8 exhibited an addition (46,XY,add(8)(q24)) in 58% and 52% of NP2/K4DT and IP4/K4DT populations, respectively. This result suggests that transduction of mutant CDK4 and cyclin D1 in combination with TERT (K4DT) sometimes induced chromosomal abnormality or enabled such cells to survive. As chromosomal abnormalities are rarely found in nasal cells, it is unlikely that these transgenes themselves induce constitutive chromosomal instability. Though high-risk HPV E6 and E7 oncoproteins are reported to independently induce numerical and structural chromosome instability^41^, HPV58-positive primary NP3 cells and 10% NP3/T cells showed normal diploid karyotype, suggesting expression levels of E6 and E7 oncoproteins in these cells were low. However, trisomy chromosome number 4 (47,XY, + 4) was found in 90% of the NP3/T cell populations. Duesberg and McCormack investigated karyotypic flexibility of primary human kidney (HA1 and HA1-2) and skin cells (BJ) after transfection with overexpressed telomerase. Their results suggested that such cells could contain clonal and flexible karyotypes^42^. In addition, an increased aneuploid cell population was observed in cen3tel cell culture at PD100 compared to PD37 (100% vs 60%) after transfection with overexpressed telomerase^42^. In human mammary epithelial cells (HMEC), increase of c-MYC oncogene expression was observed with increasing population doublings (PDs) after transduction with TERT^43^. Moreover, reduction of the cell-cycle regulator protein, p16^INK4a^, was observed in the late passages of T-HME (TERT immortalized HMEC) compared to pre-senescent HME cells, while the transcriptional activity of E2F1 increased sixfold. On the other hand, functionally normal p53 and its downstream targets were not affected by overexpression of telomerase^44^. This suggests that overexpression of oncogenes such as MYC in TERT-transduced cells might be risk factors for chromosomal instability of the cells.

Tumorigenesis is generally recognized as being associated with the accumulation of genetic alterations: various mutations, such as TP53, ERBB1, NOTCH1, HRAS, KRAS, and PIK3CA are common in human head and neck cancers^45^. Mutations are more frequent in SIP-associated squamous-cell carcinoma (SCC) than in SIP^46^. We examined our cells for common mutations reportedly involved in the pathogenesis of NP and SIP: none contained mutations in TP53, KRAS, or PIK3CA. Mutations in TP53 are frequently found in SIP and SIP-associated SCC and might be involved in malignant transformation^46^. In addition, significantly increased expression of p53 was found in SIP with dysplasia and SIP with SCC compared to normal control^47^, consistant with our result that IP4 tissue, which is SIP with mild dysplasia showed higher p53 level compared to NP tissues and normal control (Fig. 7B). The similar result was observed in IP4 and IP4/K4DT cells compared to NP cells (Fig. 7C). KRAS mutations in NP are 17% and 35% in exons 11 and 12, respectively^48^. The expression of KRAS is significantly increased in NP compared to adjacent normal tissue and associated with advanced stages of NP^48^. KRAS mutations are significantly more common in SIP with dysplasia or SCC (10%) compared to SIP without dysplasia^46^. In contrast, another finding revealed that KRAS mutations do not occur in cell lines derived from sinonasal squamous-cell carcinoma (SNSCC) tissues^49^. KRAS mutation was not observed in our IP4 and IP4/K4DT cells, or in the IP4 tissue. However, mRNA expression results revealed increased expression of KRAS in IP4 tissue and their derivatives, IP4 and IP4/K4DT cells, which were established from SIP with mild dysplasia (Fig. 7B,C). According to a previous report, EGFR is involved in many types of human tumors. EGFR mutations are frequently found in SIP (88%), mostly in the form of insertions between A767 and C775 in exon 20^50^. A mutation in EGFR was observed only in our IP4 and IP4/K4DT cells, as well as in the IP4 primary tissue. This mutation was an insertion mutation in exon 20 called P772_H773insPYNP (Table 1). It is similar to a previously reported EGFR mutation in exon 20 that was found in SNSCC4 and UM-SCC-112 cell lines derived from SIP-associated SNSCC^50^. EGFR mutations located between residues A767 and C775 affect the loop following the C-helix region of the kinase domain, resulting in constitutive activation of EGFR^51^. We experimentally revealed this EGFR mutation at P772_H773insPYNP, which results in ligand-independent activation of EGFR (Fig. 6). The previous study demonstrated that insertions in EGFR exon 20 lead to independent dimerization activation of EGFR and autophosphorylation^52^, consistent with our finding of increased expression of EGFR and p-EGFR in IP4 cells, and IP4/K4DT cells. IP4 and IP4/K4DT cells in which HPV-DNA was not found by real-time PCR. This is also consistent with a previous report which investigated the relationship between EGFR mutations and HPV infection in SIP and found they are mutually exclusive^53^, suggesting that HPV infection and EGFR mutation are important, but independent, risk factors for SIP development and progression^53^. Overexpression of EGFR was found in laryngeal squamous cell carcinoma and its expression level was significantly associated with HPV infection^54^. The E5 oncoprotein of HPV could enhance EGFR surface expression and activation, inducing cell-cycle progression^55^. This might explain the higher EGFR expression levels in our NP3 tissue and the newly established HPV58-positive cells, NP3 and NP3/T, than seen in our HPV-negative NP2 tissue and cells derived from it (Fig. 7B,C). In addition, significantly increased of mRNA and protein expression level of EGFR was found in SIP compared to normal control and the expression level was related with dysplastic change of the cell^56^, consistant with our result that higher expression of EGFR was found in both IP4 tissue and cells derived from it compared to NP tissue and NP cells, respectively (Fig. 7B,C). Our newly established cell cultures appear to exhibit less genetic alteration than do other previously established SIP-associated SNSCC cells^49,57^.

In conclusion, this study describes new primary NP and IP cell cultures with unique characteristics derived from different clinical tumor specimens. These were successfully immortalized using TERT alone for HPV 58-positive cells, or using a combination of TERT, mutant CDK4, and cyclin D1 in HPV-negative cells. These cell cultures are representative of NP and SIP tissues in both clinical and genetic characteristics. They will be good in vitro models, useful for the study of NP and SIP, as well as for preclinical testing of new therapeutic strategies for NP and SIP.

## Methods

### Patient histories and tumor characteristics

Fresh tissue samples were collected from NP and SIP patients who underwent surgical treatment at Srinagarind Hospital, Faculty of Medicine, Khon Kaen University. The written informed consent was obtained from all patients. Tissue samples were collected, and all experiments were performed in accordance with the approved guidelines of the Khon Kaen University Ethics Committee in Human Research. An overview of patient data and characteristics of cell lines are shown in Table 3.

NP2: A 19-year-old male was diagnosed with a recurrent nasal polyp grade III of the maxillary sinus with low mucosal eosinophil count (9/high-power field (HPF)). An intranasal corticosteroid and antihistamine were used before and after surgical treatment.

NP3: A 11-year-old male was diagnosed with a nasal polyp grade III of the maxillary sinus with a high mucosal eosinophil count (50/HPF). HPV58 DNA was detected in the tumor tissue. An intranasal corticosteroid and antihistamine were used before and after surgical treatment.

IP4: A 68-year-old male was diagnosed with a recurrent inverted papilloma of the nasal cavity (middle turbinate) with Krouse staging T4. Histological examination showed mild dysplasia. EGFR mutation in exon 20 was observed in the tumor tissue. No medical treatment was used before or after surgical treatment.

### Cell-line establishment

Fresh tumor tissue samples from the operating room were cut into several small fragments, that were transferred to a 60 mm cell culture dish, covered with FYAD medium (F culture medium^58^ supplemented with 10 µM Y-27632 (Selleck, USA), 500 nM A-83-01 (TOCRIS, UK) and 500 nM DMH-1 (Selleck, USA)), and incubated in 5% CO_2_ at 37 °C. Five to seven days later, outgrowths of tumor and fibroblast cells were observed. Overgrowth by fibroblasts was prevented by repeated selective trypsinization. Three primary cell cultures were obtained and named NP2, NP3 (from NP tissues), and IP4 (from SIP tissue).

### Cell proliferation

Each established cell culture was seeded at a density of 70,000 cells per well in three 6-well plates covered with FYAD medium and incubated in 5% CO_2_ at 37 °C. Cells in triplicate wells were trypsinized, stained with trypan blue and were counted for viable cells by using a haemacytometer every 24 h for 5 days. The experiment was performed in 3 independent experiments. The population-doubling time (DT) was calculated using the following formula: DT = T/ln2(Xe/Xb), when T is the incubation time in any units, Xb is the cell number at the beginning of the incubation time, and Xe is the cell number at the end of the incubation time.

### Karyotyping of cell lines

Cells grown in cell-culture flasks were treated with culture medium supplemented with 1 µg/ml of colcemid for 30 min. The cells were harvested by standard trypsinization and treated with 0.56% KCl for 10 min at 37 °C. The cells were collected by centrifugation and fixed with cold MeOH: acetic acid (3:1) solution. The cell suspension was dropped onto glass slides and dried at 60 °C. The chromosomes were stained with Giemsa stain and observed under a light microscope. A total of 50 metaphase cells were evaluated for each cell line.

### DNA extraction

DNA was extracted using a commercially available system, DNeasy Blood and Tissue kits (QIAGEN, Germany), according to the manufacturer's instructions. The extracted DNA was quantified by GAPDH amplification using SYBR-green real-time polymerase chain reaction (real-time PCR).

### HPV DNA detection and genotyping

The presence of HPV DNA in the established cell lines was investigated using GP5 + /GP6 + primers^59^ and SYBR-green real-time PCR. DNA derived from SiHa and HeLa cells was used as positive controls. Samples that were positive according to SYBR-green real-time PCR were further investigated for HPV genotype by reverse line-blot hybridization (RLBH) as previously described^60^.

### Short tandem repeat (STR) analysis

Twenty-three autosomal markers (D3S1358, vWA, D16S539, CSF1PO, D6S1043, D8S1179, D21S11, D18S51, D5S818, D2S441, D19S433, FGA, D10S1248, D22S1045, D1S1656, D13S317, D7S820, Penta E, Penta D, TH01, D12S391, D2S1338, and TPOX), a quality indicator system, and amelogenin (AMEL), sex-identification markers were amplified using the commercial VeriFiler Plus PCR Amplification Kit, according to the manufacturer’s protocol. The amplicons were genotyped by multi-capillary electrophoresis on an Applied Biosystems 3130xl Genetic Analyzer, and the allele calling was performed by the GeneMapper ID-X Software Version 1.4 (Applied Biosystems).

### Gene mutation analysis

Target DNA was amplified by PCR: 1 cycle of 98 °C for 1 min; 30 cycles of 98 °C for 10 s, 55 °C for 30 s, and 68 °C for 90 s, followed by 5 min at 68 °C and kept cold at 12 °C. The PCR products were purified with GF1-AmbiClean Kit (Vivantis, Malaysia). TP53 in exons 5–9, PIK3CA in exons 9 and 20, KRAS in exon 2 and EGFR in exons 19–20 mutations were analyzed by direct sequencing using the 3130xl Genetic Analyzer (Applied Biosystems, USA). Both strands were sequenced for confirmation. The primers are given in Table 4. The electropherograms were analyzed by CRISP-ID^61^ and the Mixed Sequence Reader^62^.

**Table 4:** Sequences of primers used in this study.

Target	F-primer sequence	R-primer sequence	References
**Sequencing primers**			
TP53-Exon5-6	5′- TCTGTCTCCTTCCTCTTCCT-3′	5′-CACTGACAACCACCCTTAAC-3′	^63^
TP53-Exon7-9	5′-CCTGCTTGCCACAGGTCT-3′	5′-GCCCCAATTGCAGGTAAAAC-3′	^63^
PIK3CA-Exon9	5′-CTGTGAATCCAGAGGGGAAA-3′	5′-ACATGCTGAGATCAGCCAAAT-3′	^64^
PIK3CA-Exon20	5′-ATGATGCTTGGCTCTGGAAT-3′	5′-GGTCTTTGCCTGCTGAGAGT-3′	^64^
KRAS-Exon2	5′-TACTGGTGGAGTATTTGATAGTG-3′	5′-CTGTATCAAAGAATGGTCCTG-3′	^49^
EGFR-Exon19	5′-GTGCATCGCTGGTAACATCC-3′	5′-TGTGGAGATGAGCAGGGTCT-3′	^65^
EGFR-Exon20	5′-ATCGCATTCATGCGTCTTCA-3′	5′-ATCCCCATGGCAAACTCTTG-3′	^65^
**PCR and RT-qPCR primers**			
GP5 + /GP6 +	5′-TTTGTTACTGTGGTAGATACTAC-3′	5′-GAAAAATAAACTGTAAATC ATATTC-3′	^59^
p53	5’-TGAAGCTCCCAGAATGCCAG-3’	5’-CAGAAGATGACAGGGGCCAG-3’	This study
PIK3CA	5’- AAATTCAGTGCAAAGGCGGC-3’	5’- CGTGTAAACAGGTCAATGGCTG-3’	
KRAS	5’-TGGTCCTGCACCAGTAATATGC-3’	5’-GCGTAGGCAAGAGTGCCTTGAC-3’	
EGFR	5’-CCAGTATTGATCGGGAGAGCCGGA-3’	5’-CTTTTCCTCCAGAGCCCGACTCGC-3’	
GAPDH	5′-TCATCAGCAATGCCTCCTGCA-3′	5’-TGGGTGGCAGTGATGGCA-3′	^60^

### Gene expression analysis

RNA was extracted using TRIzol Reagent (Invitrogen, USA), according to the manufacturer's instructions. The extracted RNA was subjected to cDNA synthesis using SuperScript III First-Strand Synthesis System (Invitrogen, USA), according to the manufacturer's instructions. mRNA expression levels of EGFR, p53, KRAS, PIK3CA, and the internal control, GAPDH were investigated by RT-qPCR (Applied Biosystems QuantStudio 6 Flex Real-Time PCR System, Thermo Fisher Scientific, USA) using specific primers shown in Table 4.

### Western-blot analysis

To determine the expression level of CDK4, cyclinD1 and cytokeratin 14, cells were cultivated in FYAD medium until 90% confluence and collected cell lysates using lysis buffer. In an EGF-treatment experiment, the cells were starved overnight in serum- and EGF-free FYAD medium, then incubated in the presence or absence of 50 ng/ml of EGF for 30 min prior to harvesting cell lysates. Protein expression of CDK4, cyclinD1, cytokeratin 14, EGFR, p-EGFR, AKT, p-AKT, MAPK and p-MAPK were investigated by western blotting. The western blot was performed as described previously^66^. Antibody against CDK4 (BD Biosciences, USA, Cat# 610,147, RRID:AB_397548), cyclinD1 (BD Biosciences, UAS, Cat# 554,180, RRID:AB_395291), EGFR (Cell Signaling Technology, USA, Cat# 2646, RRID:AB_2230881), p-EGFR (Cell Signaling Technology, USA, Cat# 2234, RRID:AB_331701), cytokeratin 14 (Covance, USA, Cat# PRB-155P, RRID:AB_292096), AKT (Cell Signaling Technology, USA, Cat# 9272S, RRID:AB_329827), p-AKT (Cell Signaling Technology, USA, Cat#9271S, RRID:AB_329825), MAPK (Cell Signaling Technology Cat# 9102, RRID:AB_330744), and p-MAPK (Cell Signaling Technology, USA, Cat# 9101, RRID:AB_331646) were used as primary antibodies. Horseradish peroxidase-conjugated anti-mouse and anti-rabbit immunoglobulins (Jackson Immunoresearch Laboratories, USA) were used as secondary antibodies. Vinculin (Sigma-Aldrich, USA, Cat# V9264, RRID: AB_10603627) was used as an internal control.

### Cell immortalization

Fragments of human mutant CDK4 (CDK4R24C: a p16^INK4A^-resistant form of CDK4), the encephalomyocarditis virus internal ribosome entry site (IRES) from pIRES2-EGFP (Clontech, USA) and human cyclinD1 were PCR-amplified and recombined with pDONR221P1-P4, pDONR221P4r-P3r, and pDONR221P3-P2, respectively by BP recombination according to the manufacturer’s instruction (Invitrogen, USA), and then recombined with CSII-CMV-RfA to generate CSII-CMV-CDK4R24C-IRES-Cyclin D1. To immortalize the nasal cells, CSII-CMV-TERT and CSII-CMV-CDK4R24C-IRES-Cyclin D1 recombinant lentiviruses were produced as previously described^40^. Recombinant viral fluid was inoculated onto the early passage of nasal cells in the presence of 4 μg/ml polybrene at the multiplicity of infection of 10. Twenty-four hours post-inoculation, cells were fed with a fresh complete medium. The infected cells were passaged until uninfected control cells had senesced (around passage 10–11).

### Statistical analysis

Expression difference between parental and their derivatives, and between treated and untreated in the same cell lines were calculated using t-test in GraphPad Prism 5. Cell proliferation and mRNA expression in tissue samples and in different cell lines were analyzed by one-way ANOVA using STATA. Differences were considered statistically significant at *p* < 0.05. Data are representative of 3 independent experiments and presented as the mean ± SD.

### Ethical approval

The study was approved by the Khon Kaen University ethics committee in human research (HE611288).

## Supplementary Information

Supplementary Information.
