# Supplementary material for: Establishment and genetic characterization of cell lines derived from proliferating nasal polyps and sinonasal inverted papillomas
Source: Sci Rep. 2021 Aug 24;11:17100. doi: 10.1038/s41598-021-96444-y (PMC8384845; doi:10.1038/s41598-021-96444-y)
Supplement: Supplementary file 1 — Supplementary Information. [file 41598_2021_96444_MOESM1_ESM.pdf]

Establishment and genetic characterization of cell lines derived from proliferating nasal polyps and sinonasal inverted papillomas

Thawaree Nukpook[a,b], Tipaya Ekalaksananan[a,b], Tohru Kiyono[c,*], Pornthep Kasemsiri[d], Watchareporn Teeramatwanich[b,d], Patravoot Vatanasapt[b,d], Surachat Chaiwiriyakul[e], Piti Ungarreevittaya[e], Jureeporn Kampan[e], Kanha Muisuk[f], Chamsai Pientong[a,b,*]

[a]Department of Microbiology, Faculty of Medicine, Khon Kaen University, Khon Kaen, Thailand

[b]HPV & EBV and Carcinogenesis Research Group, Khon Kaen University, Khon Kaen, Thailand

[c]Project for Prevention of HPV-related Cancer, Exploratory Oncology Research and Clinical Trial Center, National Cancer Center, 6-5-1 Kashiwanoha, Kashiwa, Chiba 277-8577, Japan

[d]Department of Otorhinolaryngology, Faculty of Medicine, Khon Kaen University, Khon Kaen, Thailand

[e]Department of Pathology, Faculty of Medicine, Khon Kaen University, Khon Kaen, Thailand

[f]Department of Forensic Medicine, Faculty of Medicine, Khon Kaen University, Khon Kaen, Thailand

*Corresponding author

E-mail: chapie@kku.ac.th; tkiyono@east.ncc.go.jp

Full images of Western blot in Figure 3A

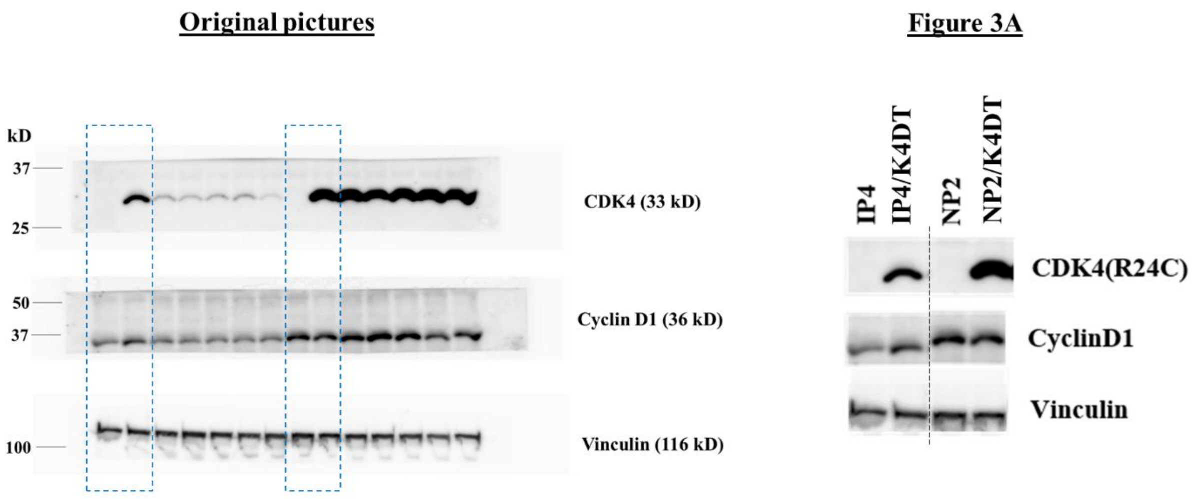

The grouping of blots in Figure 3A was cropped from the same samples loaded in 2 gels with 3 blots.

Full images of Western blot in Figure 6

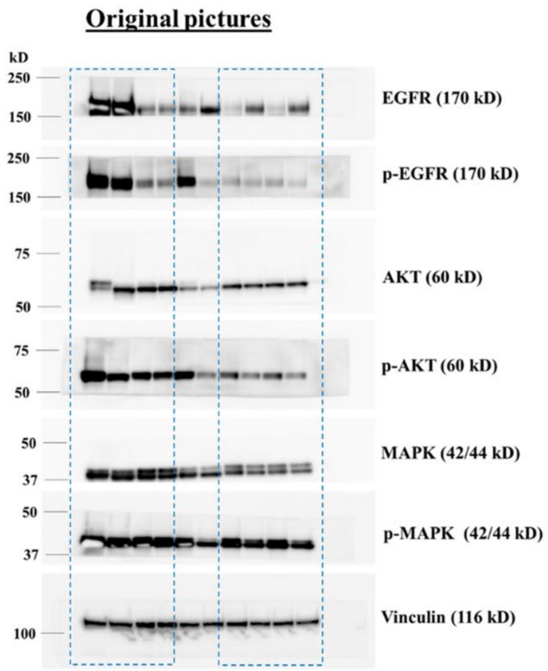

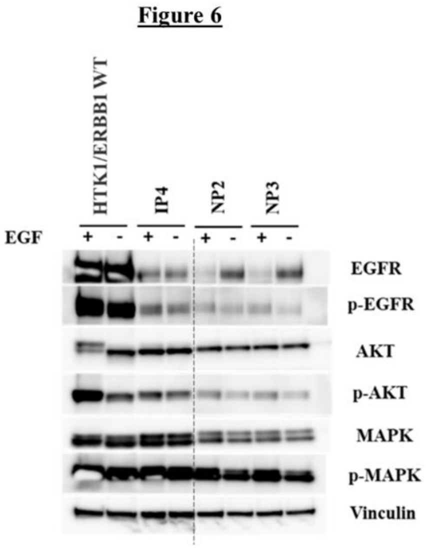

The grouping of blots in Figure 6 was cropped from the same samples loaded in 2 gels with 7 blots.

Full images of Western blot in Figure 7A

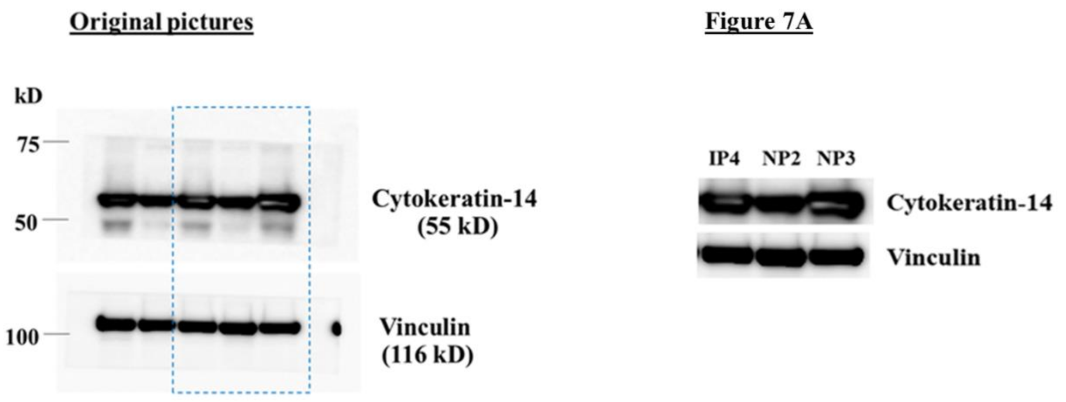

The grouping of blots in Figure 7A was cropped from the same samples loaded in a gel with 2 blots.
